# Effects of Chronic 100 mg/kg Cannabidiol Treatment in Male Double Transgenic *APP_Swe_/PS1∆E9* Mice

**DOI:** 10.3390/ph19030374

**Published:** 2026-02-27

**Authors:** Georgia Watt, Juan Olaya, Gerald Muench, Brett Garner, Tim Karl

**Affiliations:** 1 School of Medicine, Western Sydney University, Campbelltown, NSW 2560, Australia; georgia.watt@sydney.edu.au (G.W.); g.muench@westernsydney.edu.au (G.M.); 2 Neuroscience Research Australia, Randwick, NSW 2031, Australia; juan.c.olaya@outlook.com; 3 Illawarra Health and Medical Research Institute, University of Wollongong, Wollongong, NSW 2522, Australia; brettgarner30@gmail.com; 4 School of Biological Sciences, University of Wollongong, Wollongong, NSW 2522, Australia

**Keywords:** alzheimer’s disease, cannabidiol (CBD), *APP_Swe_/PS1∆E9* transgenic mice, cognition, BDNF

## Abstract

**Background/Objectives**: Alzheimer’s disease (AD) is a neurodegenerative disease for which there are no highly effective treatments, which highlights the need for novel therapeutics. Cannabidiol (CBD) has demonstrated antioxidant, anti-inflammatory and neuroprotective properties. Chronic CBD treatment (20 mg/kg and 50 mg/kg) reverses social recognition memory deficits of *APP_Swe_/PS1∆E9* (*APP/PS1*) transgenic mice; however, it does not produce effects on AD-relevant brain pathology. **Methods**: Here, we investigated whether chronic high-dose CBD treatment (i.e., 100 mg/kg intraperitoneally) in early symptomatic 7.5-month-old *APP/PS1* males would reverse cognitive deficits while also influencing neuropathological markers relevant to AD. Mice were assessed for anxiety, recognition memory, and social and aggressive behaviours before carrying out neuropathological analyses of collected brain tissue. **Results**: Vehicle-treated *APP/PS1* transgenic males demonstrated reduced aggressive behaviour and increased socio-positive behaviour. A moderate deficit in social recognition memory was restored by CBD. *APP/PS1* mice also exhibited elevated cortical proBDNF levels under vehicle treatment, and hippocampal levels of TNF-α and IL-1β were reduced in all *APP/PS1* mice. AD transgenic mice exhibited no changes in soluble or insoluble Aβ_42_ levels or PPARγ isoforms. **Conclusions**: This study found that high-dose CBD restored a moderate social recognition memory deficit. However, CBD did not have marked effects on AD-relevant neuropathological markers assessed, most likely because the AD transgenic mice were evaluated at a disease stage too early to detect significant pathological changes. Thus, the underlying mechanisms for CBD’s effect on social recognition memory require further investigation.

## 1. Introduction

Alzheimer’s disease (AD) is the predominant form of dementia—a neurodegenerative disease characterised by learning and memory deficits and non-cognitive symptoms, including social withdrawal and aggression [1]. Pathological changes are evident in the brains of people living with AD, including amyloid-β (Aβ) plaques and neurofibrillary tangles, the latter accumulating from hyperphosphorylated tau [2]. Additionally, increased levels of neurodegeneration, neuroinflammation and oxidative damage have also been associated with the pathophysiology of this disease [3]. Current options for dementia therapy are limited as they do not modify underlying disease pathology. As a result, these treatments only offer modest and temporary improvements [4]. Furthermore, recently approved AD drugs (e.g., lecanemab and aducanumab) have limited efficacy for only a select subset of patients (i.e., early-stage AD patients with elevated beta amyloid levels). These therapies are also very costly and have significant safety considerations (e.g., brain swelling or microbleeds), necessitating continual patient monitoring [5,6]. Therefore, there is an unmet clinical need for the development of effective and safe disease-modifying therapies for dementia that can be used in a broader patient population.

Targeting the endocannabinoid system can modify AD pathogenesis, including Aβ clearance, inflammation, oxidative stress and acetylcholine homeostasis [7]. Therefore, an increasing number of studies have evaluated the therapeutic potential of cannabis extracts and purified cannabinoids such as cannabidiol (CBD) for dementia. In vitro studies have found CBD to increase Aβ clearance and reduce tau hyperphosphorylation (reviewed in [8,9]). Furthermore, CBD reversed a spatial reference memory deficit in a pharmacological mouse model of AD [10].

Our own work expanded on these initial findings by utilising a double transgenic *APPswe/PS1∆9* (*APP/PS1*) mouse, which co-expresses *amyloid precursor protein* (*APP*) and *presenilin 1* (*PS1*) mutant genes. This transgenic AD mouse model demonstrates construct and face validity for AD, including age-dependent amyloid pathology and AD-relevant behavioural deficits including cognitive impairments [11,12].

Chronic treatment of the *APP/PS1* mouse model with daily intraperitoneal (i.p.) CBD doses of 20 mg/kg (early disease stages) and 50 mg/kg (late disease stages) effectively reduced cognitive deficits of the AD transgenic mice [13,14]. CBD treatment in *APP/PS1* mice also led to a moderate reduction in cortical insoluble hippocampal Aβ40 levels at later disease stages, but no changes in neuroinflammation, neurodegeneration or other AD-relevant markers were evident.

Importantly, cannabinoids, including CBD, work in a dose-dependent manner [15,16]. Therefore, the current exploratory study evaluated whether a chronic, high-dose chronic CBD treatment design (100 mg/kg) could reverse disease-relevant pathologies in *APP/PS1* transgenic mice compared to the previously published lower-dose treatment designs, as well as ameliorate AD-relevant behavioural impairments.

In this study, CBD treatment commenced in early disease stages (from around 7 months of age onwards). This is aligned with the premise that earlier treatment intervention is more likely to lead to a more pronounced modification of AD-related disease pathology [8,17]. In *APP/PS1* mice, amyloid pathology is elevated at 4 months of age, and cognitive deficits begin to appear from around 7 months of age onwards [13]. In the current study, behavioural testing of *APP/PS1* mice started after 3 weeks of treatment and included the elevated plus maze (EPM), novel object recognition task (NORT), social preference test (SPT), and resident–intruder paradigm.

Following the behavioural characterisation, established disease-relevant brain markers for AD and biological pathways associated with CBD were analysed in line with previously published work. This included brain-derived neurotrophic factor (BDNF), ionised calcium binding adaptor molecule 1 (IBA1) and the peroxisome proliferator-activation receptor γ (PPARγ) [10,18,19]. Aβ levels and neuroinflammatory markers, tumour necrosis factor-α (TNF-α) and interleukin 1β (IL-1β), were measured in the hippocampus via ELISAs as Aβ depositions are thought to start there [20,21,22], whereas all other markers were measured in the cortex using Western blots in line with our previous work [14,23].

## 2. Results

### 2.1. Anxiety/Locomotion

Two-way ANOVA for total distance travelled in the EPM revealed no difference between *APP/PS1* and WT mice and CBD treatment did not affect locomotion either [no main effects, all *p* values > 0.05] (Table 1). Similarly, there were no main effects of ‘genotype’, ‘treatment’ or ‘genotype’ by ‘treatment’ interactions on the anxiety-related parameters, percentage time spent and percentage distance travelled on the open arms (all *p* values > 0.05) (Table 1). When evaluating the first and second halves of the open arms, two-way ANOVAs confirmed there were no effects of genotype or chronic CBD treatment on percentage distance travelled or time spent on either half of the open arms (all *p* values > 0.05; Table 1).

### 2.2. Cognition

#### 2.2.1. NORT

Two-way ANOVA for percentage of time spent sniffing the novel object revealed no main effects of ‘genotype’, ‘treatment’ or ‘genotype’ by ‘treatment’ interactions (all *p* values > 0.05; Table 2).

#### 2.2.2. SPT—Sociability

**Figure 1:**
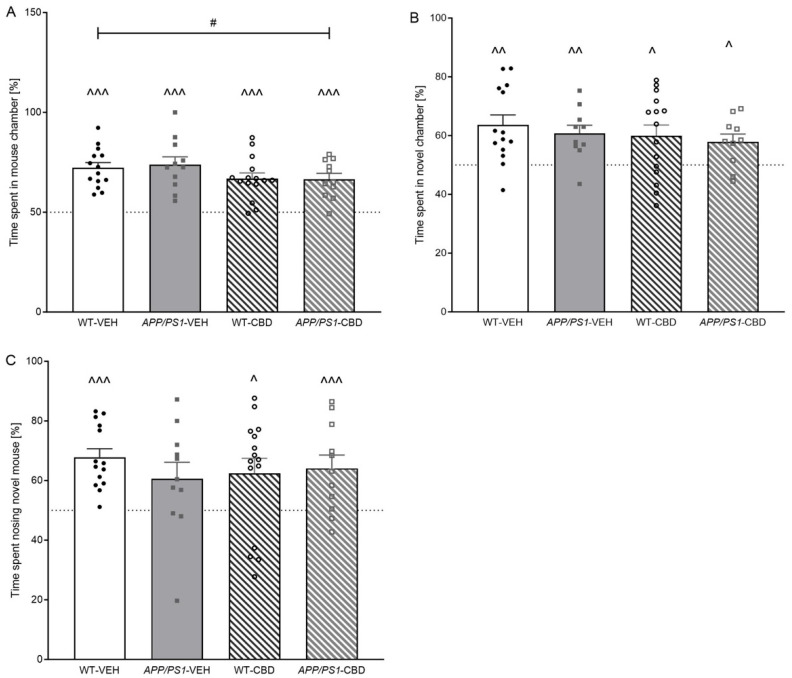
(**A**–**C**): Sociability and social recognition in the social preference test. Time spent [%] (**A**) in mouse chamber (i.e., compared to empty chamber), (**B**) in novel mouse chamber and (**C**) *sniffing* the novel mouse enclosure (i.e., compared to the chamber containing the familiar mouse) for male *APPSwe/PS1∆E9* (*APP/PS1*) transgenic mice and non-transgenic wild-type-like (WT) littermates treated with 100 mg/kg cannabidiol (CBD) or vehicle (WT-VEH *n* = 14; *APP/PS1*-VEH *n* = 11; WT-CBD *n* = 15; *APP/PS1*-CBD *n* = 11). Data are presented as means ± SEM. Two-way ANOVA ‘treatment’ effects are presented as ‘#’ (# *p* < 0.05). Significant single-sample *t*-test results against chance levels are presented as ‘^’ (^ *p* < 0.05, ^^ *p* < 0.01 and ^^^ *p* < 0.001). Dotted line indicates by chance (i.e., 50%) levels of time spent in chambers or on sniffing.

Single-sample *t*-tests revealed that all groups demonstrated an above chance preference for the mouse chamber [i.e., percentage time in mouse chamber: WT-VEH *p* < 0.0001; *APP/PS1*-VEH *p* < 0.0001; WT-CBD *p* < 0.0001; *APP/PS1*-CBD *p* < 0.0001] (Figure 1A). Interestingly, two-way ANOVA revealed a ‘treatment’ effect [F(1,47) = 4.242, *p* = 0.045] with CBD-treated mice spending less time in the mouse chamber compared to the vehicle-treated group.

#### 2.2.3. SPT—Social Novelty

Single-sample *t*-tests indicated a preference for the novel chamber in all experimental groups when assessing time spent in chamber [WT-VEH *p* = 0.002; *APP/PS1*-VEH *p* = 0.004; WT-CBD *p* = 0.016; *APP/PS1*-CBD *p* = 0.016] (Figure 1B). In addition, two-way ANOVA revealed no effect of ‘genotype’, ‘treatment’ or ‘genotype’ by ‘treatment’ interactions (all *p* values > 0.05). However, we also analysed time spent sniffing the novel mouse as a much more accurate measure of social recognition memory compared to chamber time. *APP/PS1*-VEH mice failed to demonstrate a preference for the novel mouse [*APP/PS1*-VEH: t(10) = 1.939, *p* > 0.05], whereas all other experimental groups demonstrated this preference [WT-VEH: t(13) = 6.276, *p* < 0.0001; WT-CBD: t(14) = 2.469, *p* = 0.027; *APP/PS1*-CBD: t(10) = 3.129, *p* = 0.01] (Figure 1C). Analysing for treatment and genotype effects across groups, two-way ANOVA revealed no further differences (all *p* values > 0.05).

### 2.3. Socio-Positive and Aggressive Behaviours in the Resident–Intruder Task

**Figure 2:**
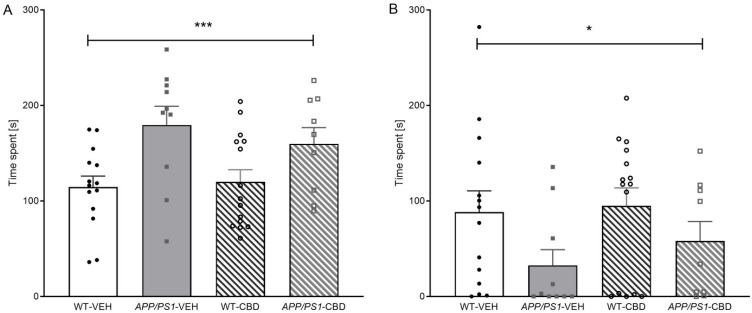
(**A**,**B**): Behaviours displayed in the resident–intruder task. Total time [s] spent engaging in (**A**) socio-positive behaviours (i.e., *anogenital sniffing*, *sniffing*, and *following*) and (**B**) aggressive behaviours (i.e., *tail rattling*, *aggressive grooming*, and *wresting*) for male *APP_Swe_/PS1∆E9 (APP/PS1*) transgenic mice and non-transgenic wild-type-like (WT) littermates treated with 100 mg/kg cannabidiol (CBD) or vehicle (WT-VEH *n* = 14; *APP/PS1*-VEH *n* = 10; WT-CBD *n* = 15; *APP/PS1*-CBD *n* = 9). Data are presented as means ± SEM. Two-way ANOVA ‘genotype’ effects are presented as ‘*’ (* *p* < 0.05 and *** *p* < 0.001).

The total time spent engaging in socio-positive behaviours (i.e., *sniffing*, *anogenital sniffing*, and *following*) was elevated in *APP/PS1* transgenic males compared to WTs [F(1,44) = 12.163, *p* = 0.001] regardless of CBD treatment (no significant ‘genotype’ by ‘treatment’ interaction; *p* > 0.05) (Figure 2A). Analysing individual behaviours, *APP/PS1* males spent significantly more time on *sniffing* [F(1,44) = 14.536, *p* < 0.0001], and this phenomenon was not affected by CBD treatment (i.e., no ‘genotype’ by ‘treatment’ interaction; *p* > 0.05). However, there was a significant ‘genotype’ by ‘treatment’ interaction [F(1,44) = 4.341, *p* = 0.043] for duration of *anogenital sniffing* (Table 3). When split by treatment, *APP/PS1*-VEH mice spent significantly longer on this behaviour compared to corresponding vehicle-treated WT mice [F(1,22) = 8.515, *p* = 0.008], whereas this genotype effect was absent in CBD-treated animals (*p* > 0.05). *APP/PS1* mice also exhibited a higher frequency of *anogenital sniffing* [F(1,44) = 4.126, *p* = 0.048] compared to WT mice, but this genotype difference was evident in both treatment groups (no ‘genotype’ by ‘treatment’ interaction, *p* > 0.05; Table 3). There was no effect of genotype or CBD treatment on *following* or *rearing* directed toward social opponents (all *p* values > 0.05; Table 3).

Interestingly, the total time spent engaging in aggressive behaviours (i.e., *wrestling*, *tail rattling*, and *aggressive grooming*) was lower in *APP/PS1* mice compared to WTs [F(1,44) = 4.908, *p* = 0.032] (Figure 2B), and a similar trend was observed for the total frequency of aggressive behaviours [F(1,44) = 4.019, *p* = 0.051]. This genotype effect was predominantly due to AD transgenic mice exhibiting significantly less *wrestling* behaviour (with respect to both time spent and frequency) than WTs [time: F(1,44) = 5.470, *p* = 0.024; frequency: F(1,44) = 4.620, *p* = 0.037—no ‘genotype’ effects for *tail rattling* or *aggressive grooming*; all *p* values > 0.05; Table 1]. CBD-treated mice spent more time *tail rattling* than vehicle-treated mice [F(1,44) = 4.877, *p* = 0.032] regardless of genotype (no ‘genotype’ by ‘treatment’ interaction, *p* > 0.05; Table 3).

### 2.4. Amyloid β

**Figure 3:**
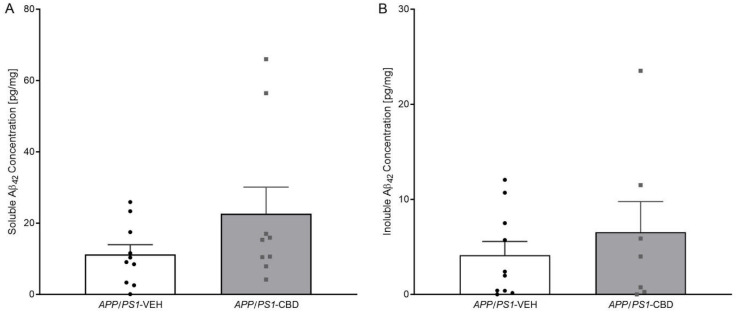
(**A**,**B**): ELISA results for Aβ_42_ in the hippocampal tissue of *APP/PS1* mice. ELISA results for (**A**) soluble Aβ_42_ and (**B**) insoluble Aβ_42_ levels for male *APPSwe/PS1∆E9* (*APP/PS1*) transgenic mice treated with 100 mg/kg cannabidiol (CBD) or vehicle (*APP/PS1*-VEH *n* = 10; *APP/PS1*-CBD *n* = 9). Data are presented as means ± SEM.

Unpaired *t*-test for hippocampal levels of soluble (Figure 3A) and insoluble Aβ_42_ (Figure 3B) revealed that there were no significant differences between vehicle- and CBD-treated *APP/PS1* mice (all *p* values > 0.05).

### 2.5. Neuroinflammation

**Figure 4:**
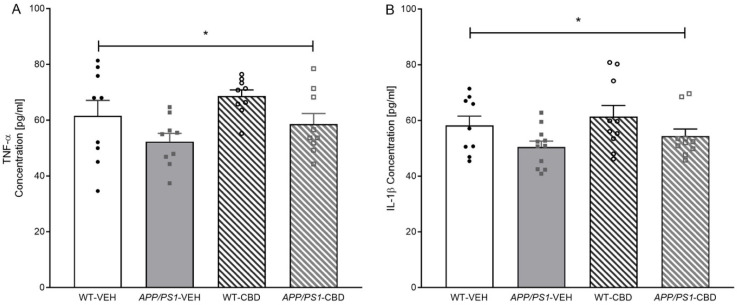
(**A**,**B**): ELISA results for TNF-α and IL-1β in the hippocampal tissue of *APP/PS1* WT mice. ELISA results for (**A**) TNF-α levels and (**B**) IL-1β levels for male *APP_Swe_/PS1∆E9* (*APP/PS1*) transgenic mice and non-transgenic wild-type-like (WT) littermates treated with 100 mg/kg cannabidiol (CBD) or vehicle (WT-VEH *n* = 9; *APP/PS1*-VEH *n* = 11; WT-CBD *n* = 10; *APP/PS1*-CBD *n* = 10). Data are presented as means ± SEM. Two-way ANOVA ‘genotype’ effects are presented as ‘*’ (* *p* < 0.05).

Two-way ANOVA for hippocampal TNF-α levels revealed a main effect of ‘genotype’ [F(1,32) = 6.329, *p* = 0.017], where *APP/PS1* mice had a lower concentration of hippocampal TNF-α compared to WT mice (Figure 4A). There was no ‘genotype’ by ‘treatment’ interaction and no significant effect of CBD on TNF-α (all *p* values > 0.05). Similarly, hippocampal IL-1β concentration was lower in *APP/PS1* transgenic compared to WT mice [F(1,36) = 5.753, *p* = 0.022], with CBD having no effect on this marker or the genotype difference (all *p* values > 0.05; Figure 4B). Testing for cortical IBA1 protein levels revealed no significant differences regardless of genotype or CBD treatment (all *p* values > 0.05; Supplementary Table S1).

### 2.6. Neurodegeneration

**Figure 5:**
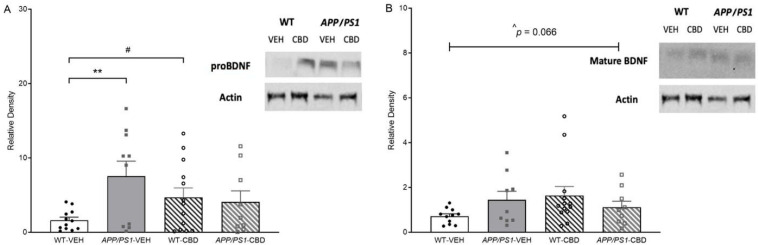
(**A**,**B**): Western blot results for neurodegeneration markers in the cortical tissue of *APP/PS1* and WT mice. Western blot results for (**A**) proBDNF and (**B**) mature BDNF levels for male *APPSwe/PS1∆E9* (*APP/PS1*) transgenic mice and non-transgenic wild-type-like (WT) littermates treated with 100 mg/kg cannabidiol (CBD) or vehicle (WT-VEH *n* = 12; *APP/PS1*-VEH *n* = 11; WT-CBD *n* = 14; *APP/PS1*-CBD *n* = 10). Data are presented as means ± SEM. Two-way ANOVA ‘treatment’ effects are presented as ‘#’ (# *p* < 0.05) and ‘genotype’ effects as ‘**’ (** *p* < 0.01). (**B**) There was also a trend toward ‘genotype × treatment’ interaction for mature BDNF levels [F(1,38) = 3.595, ^ *p* = 0.066].

Two-way ANOVA for cortical proBDNF (37 kDa) revealed a strong trend toward an overall ‘genotype’ effect [F(1,40) = 3.860, *p* = 0.056], where proBDNF was elevated in *APP/PS1* mice, and a significant ‘genotype’ by ‘treatment’ interaction [F(1,40) = 5.580, *p* = 0.02]. Split by ‘treatment’, vehicle-treated APP/PS1 mice showed increased proBDNF levels compared to respective WT males [F(1,20) = 10.158, *p* = 0.005], which was not detected in CBD-treated mice (*p* > 0.05; Figure 5A). When splitting by ‘genotype’ instead, CBD increased proBDNF levels in WT mice [F(1,23) = 4.964, *p* = 0.036] but not AD transgenic mice (*p* > 0.05).

Two-way ANOVA for cortical mature BDNF (14 kDa) revealed only a moderate trend toward a ‘genotype’ by ‘treatment’ interaction [F(1,38) = 3.595, *p* = 0.066; Figure 5B]. No significant main effects of genotype or treatment were detected (all *p* values > 0.05).

### 2.7. PPARγ Isoform Levels

Two-way ANOVA for protein levels of cortical PPARγ1 and PPARγ2 isoforms revealed no significant effect of ‘treatment’, ‘genotype’ or their interaction (all *p* values > 0.05; Supplementary Table S1).

## 3. Discussion

This study demonstrated that chronic treatment with 100 mg/kg CBD reversed moderate social recognition memory deficits in 7.5-month-old male double transgenic APP/PS1 mice. This study also found that APP/PS1 males demonstrated increased socio-positive behaviours (particularly sniffing and anogenital sniffing) and reduced aggressive behaviours (particularly wrestling) compared to WTs. Most importantly, CBD treatment reversed the duration of *anogenital sniffing* of *APP/PS1* males back to WT levels. No genotype or treatment effects were evident in anxiety or novel object recognition. In addition, elevated cortical proBDNF levels in vehicle-treated *APP/PS1* mice were not evident in CBD-treated animals as the phytocannabinoid increased proBDNF expression in WT animals. *APP/PS1* mice also exhibited reduced hippocampal levels of TNF-α and IL-1β across treatment groups. No other genotype or treatment effects were evident for any brain markers investigated.

Vehicle-treated *APP/PS1* males demonstrated a moderate social recognition memory deficit [13,24] as these mice did not develop a preference for the novel mouse. Chronic CBD treatment reversed this deficit. Previous work has found lower doses of CBD to have the same effect in 8- and 12-month-old AD transgenic males [13,14]. As all test mice exhibited intact sociability, it is unlikely that changes in social baseline behaviours affected the cognitive impairment detected. Importantly, loss of facial recognition memory and social withdrawal are commonly seen in AD patients suggesting at least partial clinical relevance of the effect of CBD on social recognition memory [1].

Increased aggression is commonly seen in AD patients [25]; thus, we assessed social and aggressive behaviours of *APP/PS1* transgenic mice. Our findings indicate that territorial aggression (i.e., *wrestling* behaviour) was reduced in our *APP/PS1* model. In line with this, an earlier study on *APP/PS1* transgenic males on the mixed background (i.e., C3H/HeN—the model used in the current study) reported reduced aggression after isolation [26]. This finding contradicts what has been seen in people living with AD [27] and may be related to effects of genetic background on mouse model phenotypes [28]. Indeed, *APP/PS1* males on a pure C57BL/6J background exhibit increased levels of aggression [29,30], although it is noted that test protocol differences known to affect aggressive phenotypes [31,32] were evident across studies (i.e., isolation prior to testing).

The resident–intruder paradigm is not traditionally utilised as an appropriate test method to investigate socio-positive behaviours. Nonetheless, it is interesting to note that selected socio-positive behaviours were elevated in *APP/PS1* males, which is contrary to what has been observed in *APP/PS1* mouse models on different backgrounds and when using more valid paradigms for the evaluation of socio-positive behaviours [33,34]. Furthermore, lower-dose CBD has been found to restore social interaction deficits [35,36,37], while 100 mg/kg CBD appeared to have no effect on overall social interaction time [15,16]. The current experiments revealed a specific CBD-induced reversal of the increased *anogenital sniffing* of AD transgenic males of the vehicle treatment condition.

The study found no genotype differences or treatment effects on anxiety levels confirming a previous study in 8-month-old AD transgenic males using 20 mg/kg CBD [13]. Although CBD has been found to have anxiolytic-like effects, these have been predominantly reported when utilising acute CBD administrations [38,39]. Notably, WT mice failed to develop a preference for the novel object, although similar NORT protocols have been effective in the past [11,12].

Brain analyses were carried out to associate behavioural findings with molecular changes. Elevated cortical proBDNF levels were detected in vehicle-treated *APP/PS1* mice, which were not evident in CBD-treated animals as the phytocannabinoids selectively increased proBDNF expression in WT animals. However, there were no genotype or treatment effects when analysing mature BDNF. While mature BDNF is associated with promoting neuronal survival, differentiation, synaptic plasticity and long-term potentiation [40], proBDNF has been found to induce apoptosis, reduce dendritic spine density and facilitate long-term depression [41]. Interestingly, elevated proBDNF levels have been reported in the brains of people living with AD [42], whereas reduced levels of mature BDNF have been reported in AD patients [43] and appear associated with impaired cognition [44]. Reduced mature BDNF levels have been detected in the hippocampus and cortex of 8- to 11-month-old *APP/PS1* males [23,45], whereas our own work in 12-month-old *APP/PS1* males found no changes in mature cortical BDNF [14]. In this context, it should also be noted that lower-dose CBD treatment (5–30 mg/kg) has previously been found to increase BDNF levels [46,47,48].

CBD treatment did not affect soluble or insoluble Aβ_42_ levels in *APP/PS1* males. Similarly, CBD-enriched extract (0.75 mg/kg) had no effect on soluble Aβ_42_ in the cortex of 6-month-old *APP/PS1* males [49]. Surprisingly, hippocampal TNF-α and IL-1β protein levels were reduced in AD transgenic males, while cortical IBA1 protein levels were unaffected. In previous work, cortical mRNA expression of TNF-α and IL-1β tended to be increased in 8-month-old *APP/PS1* males [24], and IBA1 protein expression was elevated in 12-month-old *APP/PS1* males regardless of CBD treatment [14], suggesting that changes in the neuroinflammatory signalling and microglia function may occur only at later stages of the disease. Indeed, TNF-α and IL-1β have been found to be elevated at 8–9 months in *APP/PS1* mice [50,51] as well as in AD patients [52]. The distinct findings in TNF-α and IL-1β expression across studies may be related to different methods utilised (i.e., protein versus mRNA expression). Supporting this, 9- to 15-month-old *APP/PS1* mice exhibited increased mRNA expression of cortical TNF-α and IL-1β, but this finding was not confirmed when analysing protein levels [50].

Finally, PPARγ1 and PPARγ2 protein levels were not affected in this study. However, it should be noted that PPARγ agonists have been found to have beneficial effects in AD mouse models [53,54] and in mild-to-moderate AD cases [55,56].

Importantly, only male mice were investigated as the *APP/PS1* mouse model utilised shows behavioural impairments in a sex-dependent manner, so an evaluation of treatment effects across sex is of limited value. Furthermore, *APP/PS1* males did not demonstrate a strong AD-relevant disease phenotype, which makes it difficult to determine the potential therapeutic benefit of CBD. It is possible that 7.5 months of age was too young for severe behavioural impairments and molecular changes (e.g., increase in Aβ_42_ levels or markers of neuroinflammation) to develop, although other studies have reported behavioural deficits at this age [11,13]. Another limitation of this study is the fact that the measurement of protein levels may not reflect transcriptional changes in neuroinflammatory signalling [50]. Finally, the pharmacokinetic profiling of the chosen CBD treatment design was not a focus of this study, but this has previously been described [57].

In conclusion, this is the first study evaluating the effect of high-dose CBD on behavioural impairments and AD- and CBD-relevant neuropathology in male, 7.5-month-old *APP/PS1* mice. CBD restored a moderate social recognition memory deficit and appeared to reduce cortical proBDNF levels in *APP/PS1* males. However, high-dose CBD treatment did not have marked effects on the AD-relevant neuropathological markers assessed, most likely as AD transgenic mice were evaluated at a disease stage too early to find significant pathological changes. Thus, the underlying mechanisms for this effect require further investigation.

## 4. Materials and Methods

### 4.1. Animals

Double transgenic male mice expressing chimeric mouse/human *APP* (*Mo/HuAPP695swe/Swedish mutations K595N/M596L*) and mutant human *PS1* (*PS1/∆E9*) were obtained from Jackson Laboratory (Bar Harbor, ME, USA; formerly stock no. 004462, now 034829, line 85) and maintained as double hemizygotes on a mixed C57BL/6J x C3H/HeJ background as described previously [20,21]. These AD transgenic mice do not exhibit a seizure phenotype or carry the retinal degeneration allele *Pde6brd1* evident in some other AD mouse models (e.g., *APP/PS1* transgenic mice on a congenic C57/BL6J genetic background). Male *APP_Swe_/PS1∆E9* transgenic mice (*APP/PS1*; *n* = 22) and their non-transgenic littermates (wild-type-like [WT]; *n* = 29) were bred and group-housed in independently ventilated cages (Type Mouse Version 1: Airlaw, Smithfield, Australia) at the Australian BioResources (Moss Vale, Australia). Mice were transported to mouse holding and test facilities at Western Sydney University (WSU) at approximately 10 weeks of age. At WSU they were group-housed (2–4 mice per cage) in filter top cages (1144B: Techniplast, Rydalmere, Australia) with corn cob bedding (Tecniplast Australia, Rydalmere, Australia), crinkle cut (Crink-l’Nest, Kraft) and tissues for nesting. Mice were kept in a 12:12 h light:dark schedule [light phase: white light (illumination: 124 lx), dark phase: red light (illumination: <2 lx)]. Food (Rat & Mouse Pellets, Gordon’s Specialty Stockfeeds Pty Ltd., Yanderra, Australia) and water were provided *ad libitum*. Adult, male A/J mice from Animal Resources Centre (Canning Vale, Australia) were used in SPT as stranger mice and in RI as standard opponents in line with our previous studies, as these mice exhibit a very placid phenotype [13,14]. Research and animal care were approved by WSU Animal Care and Ethics Committee (#11335 and #12905) and were in accordance with the Australian Code of Practice for the Care and Use of Animals for Scientific Purposes.

### 4.2. Drug Preparation and Administration

Pure powdered (crystalline solid) cannabidiol (CBD: 2-[(1R,6R)-3-methyl-6-prop-1-en-2-ylcyclohex-2-en-1-yl]-5-pentylbenzene-1,3-diol; CAS: 13956-29-1; >97.5% purity, <0.2% delta-9-tetrahydrocannabinol; batch number not available) from THC Pharm GmbH, Frankfurt/Main, Germany, was dissolved in equal parts Tween 80 (Sigma-Aldrich Co., St. Louis, MO, USA) and 100% ethanol (CBD and CBD working aliquots were stored at −20 °C at all times). CBD was diluted with 0.9% sodium chloride to the appropriate concentration and a final ratio of 1:1:18. Ethanol and Tween 80 made up 10% of the volume. A vehicle control solution (VEH) was prepared in the same manner but without the addition of CBD. VEH and CBD were administered at a dose of 100 mg/kg body weight (BW) via intraperitoneal (i.p.) injection using an injection volume of 10 mL/kg BW. Studies have reported that high-dose CBD does not have any toxic effects [14,15,58,59]. Male *APP/PS1* and WT were either treated with VEH or CBD daily starting 3 weeks prior to the commencement of behavioural testing (test age: 7 months ± 1 week) (WT-VEH *n* = 14; *APP/PS1*-VEH *n* = 11; WT-CBD *n* = 15; *APP/PS1*-CBD *n* = 11). Treatment continued throughout behavioural testing (total treatment duration: 6 weeks) and was given after behavioural test completion, to avoid any acute effects of CBD confounding test outcomes in line with previously published studies [13]. The BW of mice was recorded on a weekly basis.

### 4.3. Behavioural Testing

Mice were tested in several assays to detect behavioural and cognitive deficits in *APP/PS1* males [11,13] (for test age and biography, see Table 4). All tests were conducted in the first half of the light phase. An inter-test interval of at least 48 h was followed to minimise the effects of repeated testing. Equipment was cleaned with 80% ethanol between test animals (except when specified).

#### 4.3.1. Elevated Plus Maze (EPM)

EPM assesses anxiety and utilises the natural conflict of mice to explore a novel environment and to avoid brightly lit, elevated open areas [60]. The ‘+’ maze was set up as previously described [61]. Test mice were placed on the centre platform, facing an enclosed arm, and allowed to explore for 5 min. ANY-Maze^TM^ tracking software version 5 (Stoelting, Wood Dale, IL, USA) was used to record time spent and distance travelled in the open arms. Both the first and second halves of the open arm were considered for anxiety-related parameters, as the second half of the arm is more aversive to explore (being further away from the more secure centre zone and enclosed arms).

#### 4.3.2. Novel Object Recognition Task (NORT)

Object recognition memory was tested using the NORT, which is based on the innate preference of rodents for novelty [62]. The test was conducted over two days in a grey Perspex chamber (35 × 35 × 30 cm). On day 1, mice were habituated to the empty arena in a 10 min trial. On day 2, 2 × 10 min trials were conducted with an inter-trial interval (ITI) of 15 min. In the first (training) trial, test mice explored two identical objects (a toy giraffe and a toy elephant; LEGO^®^DUPLO^®^, Billund, Denmark) in the arena. In the second (test) trial, one of the objects was replaced with a novel object before the mice were allowed to explore the objects again. Objects and their locations were counterbalanced across genotypes. Time spent *sniffing* and *rearing* the objects was recorded using ANY-Maze^TM^.

#### 4.3.3. Social Preference Test (SPT)

Sociability and social recognition memory [63] were evaluated using the SPT, as described previously [61]. Test mice were isolated for 1 h prior to the start of testing. In the 5 min habituation trial, mice freely explored the apparatus. In the following sociability trial, an unfamiliar age- and gender-matched stranger mouse (i.e., male A/J mouse) was put into a mouse enclosure and placed in one of the outer chambers. The test mouse was allowed to explore the chambers again for 10 min. In the final social novelty trial, a second unfamiliar stranger mouse was placed in the chamber previously containing an empty mouse enclosure. The stranger mouse from the sociability trial was kept in its original location (familiar stranger mouse). The test mouse was allowed to explore all three chambers again for a final 10 min. The ITI was 3 min, and the test mouse was kept in the centre chamber between trials. In addition to cleaning, fresh corn cob bedding was added to the chambers prior to each new test mouse. Time spent and distance travelled in each chamber, as well as time spent *sniffing* the mouse enclosures, were recorded using ANY-Maze^TM^. *Sniffing* was recorded as a more accurate measure of social recognition memory compared to the time spent in each chamber.

#### 4.3.4. Resident–Intruder Task (RI)

The RI task was used to assess territorial aggression [64]. Prior to the start of testing, test mice were isolated in their home cage for 30 min. At the start of testing, an age- and gender-matched standard opponent (male A/J mouse, lower BW than the test mouse) and the test mouse were placed simultaneously in opposite corners of the test mouse’s home cage. The mice were then allowed to freely interact for 10 min. The duration and frequency of aggressive behaviours (i.e., *tail rattling*, *aggressive grooming*, and *wresting*) and socio-positive behaviours (i.e., *anogenital sniffing*, *sniffing*, and *following*) were recorded using ANY-Maze^TM^. Data were analysed as individual behaviours and grouped into agnostic and socio-positive behaviours, as described above. Tests were stopped when fighting escalated (defined as >10 bites or continuous fighting for >10 s) to avoid injuries (only occurred in one *APP/PS1*-VEH mouse and one *APP/PS1*-CBD mouse). No aggressive or dominant behaviour was shown by any A/J mouse during testing.

### 4.4. Biochemical Analyses

#### 4.4.1. Brain Tissue Preparation

Four days after the conclusion of behavioural tests (age range: 238 ± 16 days), mice were maintained unconscious with isoflurane, then anaesthetized and perfused with phosphate-buffered saline (PBS) transcardially. The brain was divided sagittally, and the left hemisphere was fixed in 4% paraformaldehyde for 24 h and then stored in 30% sucrose solution. The right hemisphere was dissected, and the cortex and hippocampal tissue were snap-frozen in liquid nitrogen and stored at −80 °C. Final CBD/vehicle treatment was given 24 h before euthanasia.

Frozen hippocampal tissue (12–26 mg) and cortical tissue (10–24 mg) were homogenised in 12 volumes of TBS extraction buffer [140 mM NaCl, 3 mM KCl, 25 mM Tris1M pH7.4, 2 mM 1,10 phenanthroline stock, containing 1% Igepal CA630 and Sigma protease inhibitor cocktail and 10 µM phenylmethylsulfonyl fluoride] using a Precelleys 24 homogenizer (2 × 30 s, 6000× *g*). Homogenates were centrifuged at 16,100× *g* for 30 min at 4 °C. The TBS-soluble supernatant was collected and stored at −80 °C until use. The pellet was homogenised with 10 volumes of 6.25 mM guanidine hydrochloride (gHCL) in 50 mM Tris, pH8 in Precelleys 24 homogenizer (ThermoFisher Scientific, Waltham, MA, USA: 2 × 30 s, 6000× *g*) and left in the rotary at 4 °C overnight. Homogenates were centrifuged at 16,100× *g* for 30 min at 4 °C. The gHCL-soluble supernatant (TBS-insoluble) was collected and stored at −80 °C. Protein was quantified using Qubit protein assay kits from ThermoFisher Scientific.

#### 4.4.2. ELISA

In our analysis of Aβ pathology, we only investigated Aβ_42_ levels, as it has been reported to be the more pathogenic form of the protein [2]. The concentration of Aβ_42_ protein in TBS-soluble and gHCL-soluble hippocampal fractions of *APP/PS1* mice was quantified using BetaMark^TM^ β-Amyloid x-42 ELISA Kit (TMB) (Australian Biosearch, Wangarra, Australia). The concentrations of TNF-α and IL-1β protein in TBS-soluble hippocampal fractions of *APP/PS1* and WT mice were quantified using Invitrogen TNF-α Mouse ELISA Kit (ThermoFisher Scientific) and Invitrogen IL-1β ELISA Kit (ThermoFisher Scientific). Outliers were defined as ±2 standard deviations from the mean (soluble Aβ_42_: CBD *n* = 1; insoluble Aβ_42_: VEH *n* = 1, CBD *n* = 1).

#### 4.4.3. Western Blotting

Both proBDNF and mature BDNF were analysed with cleavage of proBDNF forming mature BDNF, the biologically active compound [44]. In addition, we analysed two isoforms of PPARγ, PPARγ1 and PPARγ2, as they have distinct depositions in the brain, with PPARγ2 being predominantly expressed in adipose tissue and PPARγ1 being more ubiquitously expressed [65]. TBS-soluble cortical fractions were analysed by SDS-PAGE Western blotting using antibodies: IBA1 [5 µg of protein per lane, 1:1000, Novachem (Calgary, AB, Canada) (019-19741), band size: ~14 kDa], BDNF [20 µg of proteins per lane, 1:1000, Abcam (Cambridge, UK) (ab108319), band 1 (proBDNF) size: ~37 kDa, band 2 (mature BDNF) size: ~14 kDa], PPARγ [2 µg of protein per lane, 1:2000 MilliporeSigma (Burlington, MA, USA) (ABN1445), band 1 (PPARγ2 isoform) size: ~60 kDa, band 2 (PPARγ1 isoform) size: ~55 kDa] and housekeeper antibody: actin [1:1000, Sigma-Aldrich (A2066)]. PPARγ1 and PPARγ2 were detected by one antibody and therefore, run on one gel, as were proBDNF and mature BDNF. Goat anti-rabbit IgG HRP-conjugated secondary antibody [1:2000, Millipore (AP132P)] and enhanced chemiluminescence were used to detect signals. Signals were quantified using image J software version 1.51. Data were normalised to actin levels and internal control and expressed as relative values.

### 4.5. Statistical Analysis

Statistical analyses were conducted using SPSS 25 for Mac. Behavioural data from the EPM and the RI test, as well as molecular data (except for Aβ_42_ protein levels), were analysed using two-way analysis of variance (ANOVA) for the main effects of ‘genotype’, ‘treatment’ and potential interactions. Performance in NORT and SPT was also assessed using single-sample *t*-tests to investigate if time spent *sniffing* a novel object (NORT) and time spent in chamber and *sniffing* a mouse (SPT) were greater than chance (50%). This statistical approach is in line with previous publications [11,13,14]. Unpaired *t*-tests were used to evaluate the effect of CBD treatment on Aβ_42_ levels in *APP/PS1* mice.

Differences were regarded as statistically significant if *p* < 0.05. F-values and degrees of freedom are presented for ANOVAs. Data are shown as means ± standard error of means (SEM). Significant genotype effects are denoted by ‘*’ (* *p* < 0.05, ** *p* < 0.01, *** *p* < 0.001), treatment effects are indicated by ‘#’ (^#^ *p* < 0.05, ^##^ *p* < 0.01, ^###^ *p* < 0.001) and significant ‘genotype by treatment’ interactions are denoted by ‘+’ (^+^ *p* < 0.05, ^++^ *p* < 0.01, ^+++^ *p* < 0.001). Significant *t*-test results are shown by ‘^’ (^ *p* < 0.05, ^^ *p* < 0.01, ^^^ *p* < 0.001). Trends are reported for 0.05 ^3^*p* < 0.07. A comprehensive list of detailed statistical outcomes is presented in Supplementary Table S2.

## Figures and Tables

**Table 1:** Elevated plus maze behaviours.

Treatment	Vehicle		CBD	
Genotype	WT	*APP/PS1*	WT	*APP/PS1*
Total distance travelled [m]	7 ± 1	7 ± 0	7 ± 0	7 ± 1
Distance travelled in open arms [%]	17 ± 3	20 ± 5	15 ± 3	19 ± 3
Time spent in open arms [%]	31 ± 6	31 ± 7	26 ± 5	33 ± 5
Time spent in first half of open arms [%]	23 ± 4	19 ± 4	21 ± 4	24 ± 4
Time spent in second half of open arms [%]	5 ± 2	9 ± 3	3 ± 1	7 ± 2

Locomotion and anxiety-related behaviours in the EPM for male *APP_Swe_/PS1∆E9* (*APP/PS1*) transgenic mice and non-transgenic wild-type-like (WT) littermates treated with 100 mg/kg cannabidiol (CBD) or vehicle (WT-VEH *n* = 14; *APP/PS1*-VEH *n* = 11; WT-CBD *n* = 15; *APP/PS1*-CBD *n* = 11). Data are presented as mean ± SEM.

**Table 2:** Novel object recognition memory.

Treatment	Vehicle		CBD	
Genotype	WT	*APP/PS1*	WT	*APP/PS1*
Time spent *sniffing* novel object [%]	54 ± 4	50 ± 6	50 ± 3	51 ± 4

Time spent *sniffing* novel object for male *APP_Swe_/PS1∆E9* (*APP/PS1*) transgenic mice and non-transgenic wild-type-like (WT) littermates treated with 100 mg/kg cannabidiol (CBD) or vehicle (WT-VEH *n* = 12; *APP/PS1*-VEH *n* = 9; WT-CBD *n* = 12; *APP/PS1*-CBD *n* = 8). Data are presented as mean ± SEM.

**Table 3:** Resident–intruder behaviours.

Treatment	Vehicle		CBD	
Genotype	WT	*APP/PS1*	WT	*APP/PS1*
**Time Spent [s]**				
*Sniffing* ***	84 ± 9	125 ± 16	80 ± 7	124 ± 14
*Anogenital Sniffing* ^+^	29 ± 4	52 ± 8 ***	35 ± 6	34 ± 5
*Following*	2 ± 1	3 ± 1	5 ± 2	2 ± 1
*Wrestling* *	60 ± 18	20 ± 13	65 ± 13	32 ± 13
*Tail Rattling* ^#^	3 ± 1	1 ± 1	5 ± 1	5 ± 2
*Aggressive Grooming*	25 ± 7	11 ± 6	25 ± 7	21 ± 9
*Rearing*	6 ± 1	5 ± 2	5 ± 1	6 ± 2
**Frequency [*n*]**				
*Sniffing*	41 ± 3	43 ± 4	37 ± 3	46 ± 3
*Anogenital Sniffing* *	15 ± 2	21 ± 3	14 ± 2	18 ± 2
*Following*	1 ± 0	2 ± 0	2 ± 1	2 ± 1
*Wrestling* *	20 ± 6	7 ± 5	19 ± 5	10 ± 4
*Tail Rattling*	4 ± 1	2 ± 1	6 ± 1	5 ± 2
*Aggressive Grooming*	10 ± 2	4 ± 2	8 ± 3	7 ± 2
*Rearing*	5 ± 1	3 ± 1	3 ± 0	4 ± 1

Socio-positive and aggressive behaviours (indicated in italics) in the resident–intruder task for male *APP_Swe_/PS1∆E9* (*APP/PS1*) transgenic mice and non-transgenic wild-type-like (WT) littermates treated with 100 mg/kg cannabidiol (CBD) or vehicle (WT-VEH *n* = 14; *APP/PS1*-VEH *n* = 10; WT-CBD *n* = 15; *APP/PS1*-CBD *n* = 9). Data are presented as mean ± SEM. A two-way ANOVA ‘genotype × treatment’ interaction is presented as ‘+’ (^+^ *p* < 0.05), ‘genotype’ effects are presented as ‘*’ (* *p* < 0.05 and *** *p* < 0.001) and the main ‘treatment’ effect is indicated by ‘#’ (^#^ *p* < 0.05).

**Table 4:** Test biography and age at testing.

Treatment	Days of Treatment	Vehicle		CBD	
Genotype		WT	*APP/PS1*	WT	*APP/PS1*
Age at start of treatment	0	194 ± 3	197 ± 5	196 ± 3	198 ± 5
Elevated plus maze	21	219 ± 3	223 ± 3	220 ± 3	228 ± 1
Novel object recognition task	23	223 ± 2	226 ± 3	223 ± 2	231 ± 1
Social preference test	26	228 ± 2	231 ± 3	229 ± 2	236 ± 1
Resident–intruder task	32	231 ± 3	235 ± 3	232 ± 3	240 ± 1
Tissue collection	34	235 ± 3	239 ± 3	236 ± 3	244 ± 1

Age [days] of male *APP_Swe_/PS1∆E9* (*APP/PS1*) transgenic mice and non-transgenic wild-type-like (WT) littermates treated with 100 mg/kg cannabidiol (CBD) or vehicle throughout behavioural testing. Ages are presented as mean ± age range.

## Data Availability

The original contributions presented in this study are included in the article/Supplementary Material. Further inquiries can be directed to the corresponding author.

## Supplementary Materials

The following supporting information can be downloaded at https://www.mdpi.com/article/10.3390/ph19030374/s1, Table S1: Western blot results for IBA1 and PPARγ isoforms; Table S2: Statistical outcomes for all behavioural and molecular tests.
