# Supplementary material for: Effects of Chronic 100 mg/kg Cannabidiol Treatment in Male Double Transgenic APPSwe/PS1∆E9 Mice
Source: Pharmaceuticals (Basel). 2026 Feb 27;19(3):374. doi: 10.3390/ph19030374 (PMC13029102; doi:10.3390/ph19030374)
Supplement: Supplementary file 1 [file pharmaceuticals-19-00374-s001.zip › pharmaceuticals-4120053-supplementary.pdf]

Supplementary Materials: Effects of Chronic 100 mg/kg Cannabidiol Treatment in Male Double Transgenic *APP$_{Swe}$/PS1ΔE9* Mice

Georgia Watt, Juan Olaya, Gerald Muench, Brett Garner and Tim Karl

Supplementary Table S1. Western blot results for IBA1 and PPARγ isoforms.

Treatment	Vehicle		CBD	
**Genotype**	**WT**	***APP/PS1***	**WT**	***APP/PS1***
IBA1	1.2 ± 0.2	1.3 ± 0.2	1.5 ± 0.3	1.9 ± 0.6
PPARγ1	0.6 ± 0.2	0.6 ± 0.1	0.4 ± 0.1	0.4 ± 0.1
PPARγ2	0.8 ± 0	0.9 ± 0.1	0.7 ± 0.1	0.8 ± 0.1

Cortical protein levels of IBA1 and PPARγ isoforms for male *APP$_{Swe}$/PS1ΔE9 (APP/PS1)* transgenic mice and non-transgenic wild type-like (WT) littermates treated with 100 mg/kg cannabidiol (CBD) or vehicle (WT-VEH n = 13; *APP/PS1*-VEH n =11; WT-CBD n = 13; *APP/PS1*-CBD n = 10). Data are presented as mean ± SEM.

Supplementary Table S2. Statistical outcomes for all behavioural and molecular tests.

	**Genotype (*p* value)**	**Treatment (*p* value)**	**Genotype x Treatment (*p* value)**
	**Elevated plus maze**		
Total distance travelled [m]	0.553	0.932	0.747
Distance in the open arms [%]	0.482	0.415	0.887
Time spent in the open arms [%]	0.679	0.592	0.676
Time spent in the first half of the open arms [%]	0.339	0.761	0.813
Time spent in the second half of the open arms [%]	0.077	0.123	0.193
	**Novel object recognition task**		
Time spent *nosing* the novel object [%]	0.833	0.389	0.849
	**Social preference test**		
Time spent in mouse chamber [%]	0.835	**0.045**	0.748
Time spent in novel mouse chamber [%]	0.47	0.348	0.909
Time spent *nosing* the novel mouse [%]	0.548	0.829	0.338
	**Resident-intruder task**		
Total time spent interacting with standard opponent [s]	0.701	0.601	0.903
Time spent exhibiting aggressive behaviours [s]	**0.032**	0.445	0.65
Time spent exhibiting socio-positive behaviours [s]	**0.001**	0.638	0.406
Time spent *sniffing* [s]	**< 0.0001**	0.869	0.883
Time spent *anogenital sniffing* [s]	0.059	0.3	**0.043**
Time spent *following* [s]	0.677	0.287	0.079
Time spent *wrestling* [s]	**0.024**	0.614	0.828
Time spent *tail rattling* [s]	0.631	**0.032**	0.413
Time spent *aggressive grooming* [s]	0.247	0.493	0.509
Frequency of *sniffing* [n]	0.115	0.92	0.381
Frequency of *anogenital sniffing* [n]	**0.048**	0.356	0.713
Frequency of *following* [n]	0.677	0.287	0.341
Frequency of *wrestling* [n]	0.037	0.792	0.714
Frequency of *tail rattling* [n]	0.397	0.076	0.504
Frequency of *aggressive grooming* [n]	0.127	0.679	0.362

**Neuroinflammation-relevant markers**			
TNF-α hippocampal levels	**0.017**	0.091	0.919
IL-1β hippocampal levels	**0.022**	0.254	0.909
IBA1 cortical levels	0.472	0.31	0.188
PPARγ1 cortical levels	0.965	0.074	0.789
PPARγ2 cortical levels	0.273	0.19	0.951
**Neurodegeneration-relevant markers**			
proBDNF cortical levels	0.056	0.883	**0.02**
mature BDNF cortical levels	0.756	0.378	0.066

P-values for all two-way ANOVAs conducted for behavioural tests, western blots and ELISAs for male *APP_{Swe}/PS1ΔE9 (APP/PS1)* transgenic mice and non-transgenic wild type-like (WT) littermates treated with 100 mg/kg cannabidiol (CBD) or vehicle are shown; significant results ($p < 0.05$) are highlighted in bold.
